# Alte und neue Perspektivierungen auf die Arzneimittelforschung. Aus den Studien zur Praxis der Arzneimittelerprobung an Heimkindern und Anstaltsbewohner:innen von 1945 bis 1975

**DOI:** 10.1007/s00048-024-00389-y

**Published:** 2024-05-27

**Authors:** Marion Hulverscheidt

**Affiliations:** Fachbereich 05, Gesellschaftswissenschaften, Universität Kassel, Nora-Platiel-Straße 5, 34109 Kassel, Deutschland

Kaminsky, Uwe und Katharina Klöcker 2020: *Medikamente und Heimerziehung am Beispiel des Franz Sales Hauses. Historische Klärungen – Ethische Perspektiven*. Münster: Aschendorff 2020, geb., 288 S., 36,00 €, ISBN: 978-3-402-24697‑9.

Sparing, Frank 2020: *Medikamentenvergabe und Medikamentenerprobung an Kindern und Jugendlichen. Eine Untersuchung zu kinder- und jugendpsychiatrischen Einrichtungen des Landschaftsverbandes Rheinland 1953 bis 1975*. Berlin: Metropol, brosch., 140 S., 16,00 €, ISBN: 978-3-86331-531‑3.

Meier, Marietta, Mario König und Magaly Tornay 2019: *Testfall Münsterlingen. Klinische Versuche in der Psychiatrie, 1940–1980.* Zürich: Chronos, geb., 336 S., 30 Abb., 38,00 €, ISBN: 978-3-0340-1545‑5, 2. Auflage 2020.

Lenhard-Schramm, Niklas, Dietz Rating und Maike Rotzoll 2022: *Göttliche Krankheit, kirchliche Anstalt, weltliche Mittel. Arzneimittelprüfungen an Minderjährigen im Langzeitbereich der Stiftung Bethel in den Jahren 1949 bis 1975.* (Schriften des Instituts für Diakonie- und Sozialgeschichte an der Kirchlichen Hochschule Bethel, 36) Bielefeld: Verlag für Regionalgeschichte, geb., 288 S., 40 s/w-Abbildungen, 16 Farbabbildungen, 63 Tabellen, 16 Diagramme, 24,00 €, ISBN: 978-3-7395-1306‑5.

## Hinführung

Im August 2021 wurde das Sommerloch durch einen Skandal gefüllt: Dem Bonner Kinder- und Jugendpsychiater Michael Winterhoff, bekannt durch Bestseller zum Thema Kinder und Erziehung, wurde vorgeworfen, Kindern und Jugendlichen in Einrichtungen der Jugendhilfe über Jahre hinweg und in zu hoher Dosis das Neuroleptikum Pipamperon verordnet zu haben, um ihre vermeintlich gestörte emotionale Erreichbarkeit zu verbessern (Rosenbach 2023).

Das Skandalon umfasst dabei mehrere Ebenen:

1. Einem medial präsenten Arzt wird vorgeworfen, zu viele Medikamente und zudem Psychopharmaka zu verschreiben, welche bei Kindern nicht indiziert sind;
2. Ein Arzt, der sich mit kranken Kindern beschäftigt, erlaubt sich ein Urteil über die gesamtgesellschaftliche Situation in der Erziehung von Kindern und Jugendlichen; und
3. Trotz staatlicher Kontrolleinrichtungen bleibt öffentlich unsichtbar, was in Heimen und Einrichtungen der Kinder- und Jugendhilfe geschieht; die vermeintlichen Schutzräume werden so zu Orten der Bedrohung.

Die Debatte um Michael Winterhoff kann aber nur als eine Episode in der immer wieder problematisierten psychiatrisch-klinischen Praxis gegenüber vulnerablen Gruppen gesehen werden. Arzneimittelgaben jenseits der intentionalen Symptomlinderung oder individuellen Heilung von Patient:innen standen häufiger im Fokus der Kritik, zumal, wenn es um die Ruhigstellung oder Bestrafung von Schutzbefohlenen ging oder die Erprobung noch nicht zugelassener Arzneistoffe. Dieser Review-Essay versucht, die rezenten Ergebnisse der sozialhistorischen Aufarbeitung von Arzneimittelgaben vor allem an Kinder und Jugendliche zwischen 1945 und den 1970er Jahren zu überblicken und mit der Frage nach einem weiteren Gewinn für die Wissenschaftsgeschichte gegen den Strich zu bürsten. Aus wissenschaftshistorischer Position heraus ist die „Fortschrittsgeschichte“ der Arzneimitteltherapie im 20. Jahrhundert (Weber 1999; Shorter 2003) mittlerweile einer kritischeren Betrachtung gewichen, die insbesondere die Patient:innen und ihr Handeln deutlicher in den Blick nimmt (Balz 2010; Tornay 2016; Hess et al. 2016). Medikamentenerprobung oder Arzneimittelversuche gehören zur wissenschaftlichen Praxis, Prüfungen auf Wirksamkeit und Verträglichkeit und klinische Erprobungen sind Teil der Entwicklung neuer Wirkstoffe. Sie knüpften in der paternalistischen, von männlichen Ärzten und Kliniksleitern dominierten Atmosphäre der 1950er und 1960er Jahre – potenziert durch medizinische Fortschritte – das Netz, durch das die medikamentöse Therapie abgesichert und in letzter Konsequenz am Menschen durchgeführt wurde. Es galt und gilt, den unauflösbaren Zielkonflikt der Medizin – die Aporie zwischen wissenschaftlichem Erkenntnisinteresse und ärztlicher Hilfestellung in der Praxis – auszubalancieren. Cathy Gere hat 2017 mit großer Verve für den angloamerikanischen Raum beschrieben, wie historisch kontingent das Balancieren zwischen diesen Polen war (Gere 2017). Die besondere Situation von Kindern in der Medikamentenerprobung ist durch dreierlei Ebenen gekennzeichnet: der Ebene der Zustimmungsfähigkeit, der Ebene der Spezifität und der Ebene der Zugriffsmöglichkeit. Es stellt sich also die Frage, inwieweit Kinder als einwilligungs- und zustimmungsfähig galten und wie dies bestimmt werden konnte. Wurden Medikamente an Kindern erprobt, weil sie für diese zugelassen werden sollten (Unterschied zwischen Heilung und Erprobung zur Dosisfindung) oder wurde der einfachere Zugriff auf Kinder – insbesondere in Heimen und Anstalten – mit einem stabilen Beobachtungsrahmen (alle essen dasselbe, der Tagesablauf ist in der Struktur bei allen gleich) als Argument dafür angeführt, um an diesen bislang „nutzlosen“ Mitgliedern der Gesellschaft neue Erkenntnisse zu generieren?

Als Ausgangsort für den jüngsten medialen Aufschrei zu Medikamentengaben an Kindern und Jugendlichen gilt der „Runde Tisch Heimkinder“ (RTH), der 2010 seinen Bericht über die Missstände in der Heimerziehung in der BRD zwischen 1945 und 1970 vorlegte (Arbeitsgemeinschaft für Kinder- und Jugendhilfe 2010). Darin erwähnen Berichte ehemaliger Heimkinder Medikamentengaben zum Zweck der Ruhigstellung kursorisch, es gab jedoch Mittel und Expertisen, hier weitere Nachforschungen anzustellen. Die besondere Situation von Heimkindern zeichnet sich dadurch aus, dass sie in der Entwicklung begriffen und damit besonders vulnerabel sind und zum zweiten juristisch keine Möglichkeit der Zustimmung hatten. Diese wäre nur über die Erziehungsberechtigten beziehungsweise über die Sorgeberechtigten – also die Anstaltsleitung – einzuholen. Außerdem waren sie durch den Status als Heimkinder bereits stigmatisiert, weil sie als behindert, entwicklungsverzögert, verwahrlost etc. galten.

Zwar hatte bereits Asmus Finzen, Psychiater und Aktivist in der Psychiatrie-Enquête 1969, über Studien mit Medikamenten an Kindern und Jugendlichen berichtet, bei denen er die Freiwilligkeit der Zustimmung als fraglich ansah (Finzen 1969: 129–133), ein öffentliches Echo fand dies jedoch nicht. Eine ethische Reflexion hinsichtlich der Zustimmungsfähigkeit von Kindern sowohl nach Alter als auch nach Bildungsgrad soll und kann hier nicht vorgenommen werden. Es sei angemerkt, dass die Einschätzung der Fähigkeit von Kindern sich auch im 20. Jahrhundert wandelte, ebenso wie die Absolutsetzung der Einwilligung der Erziehungsberechtigten. Im Jahr 2011 streifte Uwe Kaminsky die Thematik der Arzneimittelerprobungen an Kindern in einem Versuch mit dem Neuroleptikum Chlorprothixen, das den Bewohner:innen im diakonischen Heim Neu-Düsselthal probeweise verabreicht wurde (Kaminsky 2011: 488). Dadurch sowie durch den RTH angeregt, suchte die Pharmazeutin Sylvia Wagner in Fachzeitschriften nach Hinweisen auf Medikamentenerprobungen an Kindern und Jugendlichen und fragte nach personalen Kontinuitäten aus der Zeit des Nationalsozialismus und nach vorliegenden Einwilligungen der Proband:innen – letztere fand sie so gut wie gar nicht. Wagners Veröffentlichung im Jahr 2016 löste große mediale Aufmerksamkeit aus, was eine aufarbeitende Forschung nach sich zog, beauftragt vor allem vonseiten der Einrichtungen und ihrer öffentlichen Aufsichtsbehörden (Wagner 2016). Im Zentrum der Kritik standen nämlich diese und nicht etwa die Ärzt:innen, die die Medikamente verabreicht hatten, oder die Pharmafirmen, galten sie doch als diejenigen, die einer Teilnahme an den Erprobungen zugestimmt hatten – eben als stellvertretende Erziehungsberichtigte oder Verantwortliche. Medikamentenerprobungen gelten bereits per se als moralisch verwerflich, zumal an Kindern oder Menschen, die nicht nach ihrer Einwilligung hierzu gefragt werden (können). Gemäß den rechtlichen Regelungen der damaligen Zeit waren sie jedoch legal. So forderte die Nordrhein-Westfälische Landesregierung weitere Aufklärung, da den Betroffenen damals „durch die medizinisch nicht indizierte Verabreichung von Arzneimitteln schweres Leid zugefügt“ worden sei, so der nordrhein-westfälische Gesundheitsminister Laumann (Rutz 2020). Die Veröffentlichungen von Frank Sparing sowie von Uwe Kaminsky und Katharina Klöcker wurden aus dieser Forderung heraus angestoßen und beziehen sich auf Einrichtungen in Nordrhein-Westfalen, ebenso wie die Studie von Niklas Lenhard-Schramm, Dietz Rating und Maike Rotzoll zur Erprobung von Antiepileptika und Psychopharmaka in der Anstalt Bethel der von Bodelschwinghschen Stiftung. Für Niedersachsen und Schleswig-Holstein liegen ebenso Berichte vor (Hähner-Rombach & Härtel 2019; Beyer et al. 2021). Aus dem bundesweiten Projekt „Leid und Unrecht“, der Begleitforschung zur Entschädigung von Heimkindern, ist im Jahr 2021 ein umfassender Bericht erschienen, in dem versucht wurde, die lokalen und regionalen Einzelstudien zu einem nationalen Gesamtbild zusammenzuführen (Fangerau et al. 2021).

## Die Studien

Frank Sparing beschreibt detailliert die Versuche mit den Neuroleptika Pipamperon und Haloperidol an Kindern zwischen 8 und 15 Jahren, die in Rheinischen Landeskliniken – unter anderem in der Landesklinik für Jugendpsychiatrie Süchteln bei Viersen am Niederrhein – untergebracht waren. Speziell diese Klinik besaß Modell- und Vorzeigecharakter für die moderne kinder- und jugendpsychiatrische Versorgung. Kinder kamen sowohl zur Beobachtung und Begutachtung als auch zur Dauerpflege hierher. Anhand einer Analyse einer Zufallsstichprobe von Krankenakten sowie den publizierten Forschungsergebnissen kann Sparing eine Periodisierung erkennen. Wurden Psychopharmaka zunächst mit großer Zurückhaltung verabreicht, nahm diese Einflussmöglichkeit in den 1970er Jahren nach dem Erscheinen von Lehrbüchern und mehreren wissenschaftlichen Fachartikeln an Fahrt auf. Um die Kosten dieser Arzneimitteltherapie zu reduzieren, wurde es als gangbarer Weg angesehen, auf Prüfpräparate der Pharmaindustrie zurückzugreifen. Das damit einhergehende Risiko nahmen die Ärzt:innen für ihre Schutzbefohlenen in Kauf. Sparing beschreibt eindrücklich die positive Wirkung der Arzneimittel: Patient:innen wurden für psycho- und soziotherapeutische Interventionen zugänglich, weniger Fixierungen und entwürdigende Sicherungen schienen notwendig. Angeregt durch das Max-Planck-Institut für Psychiatrie in München begannen Anfang der 1960er Jahre in den Rheinischen Landeskrankenhäusern Planungen, sich verstärkt der Forschung zuzuwenden, um sich auf diesem Gebiet zu profilieren. Dass das „Beobachtungsgut“ insbesondere in einer ungestörten Langzeitbeobachtung so gut zu kontrollieren war, wurde als stärkendes Argument angeführt. Realisiert wurde das Forschungsprogramm des Landesverbandes Rheinland nicht im Großen, Einzelprojekte wurden durchgeführt, ohne dass Skrupel bestanden, Arzneimittelerprobungen an Kindern und Jugendlichen durchzuführen. Sparing hinterfragt ebenfalls, ob die Versuche tatsächlich Erkenntnisse brachten, die für geistig behinderte und psychisch kranke Kinder von Nutzen waren oder eher der Ruhigstellung und somit der Entlastung des Klinikpersonals dienten (Sparing 2020: 121). In Süchteln sollte die Verabreichung von Neuroleptika und Beruhigungsmitteln von den 1960ern über die 70er Jahre bis in die 80er Jahre stark zurückgehen und zunehmend durch pädagogische Interventionen ersetzt werden.

Der Diakoniehistoriker Uwe Kaminsky und die Ethikerin Katharina Klöcker beschäftigen sich in ihrer Untersuchung mit der Frage nach dem Einsatz von Medikamenten in der Heimerziehung am Beispiel des Franz Sales Hauses, das als katholische Einrichtung in Essen für Kinder und Jugendliche mit geistiger Behinderung rund 700 Menschen ein Zuhause in der Fürsorgeerziehung bot. Kaminsky zog Verwaltungsakten, Patient:innenakten, Akten aus dem Firmenarchiv von Merck sowie Interviews mit Zeitzeug:innen für seine historische Analyse heran. Er konnte so nicht nur ermitteln, welche Medikamente verabreicht wurden, sondern auch, wer diese Arzneien bekam und wie dies begründet wurde. In mehr als der Hälfte der Krankenakten ließen sich Hinweise auf eine pharmakologische Behandlung finden. Es wurden Neuroleptika, Antidepressiva und Tranquilizer verabreicht als auch Sedativa und Mittel gegen Epilepsie, obwohl eine diesbezügliche Diagnose nicht in allen Einzelfällen vorlag. Diese Medikamente wurden zur Ruhigstellung und als Strafe eingesetzt. Das Neuroleptikum Decentan (Perphenacin der Firma Merck) beispielsweise wurde bereits vor seiner Zulassung (1957) vom Anstaltsarzt Waldemar Strehl verabreicht. Für diese Arzneimittelerprobung wurde keine Zustimmung seitens der Patient:innen eingeholt, es finden sich auch keine dokumentierten Aufklärungen oder informierte Zustimmungen. Strehl hat dieses Mittel teilweise überdosiert, wobei es zu starken Nebenwirkungen wie Krämpfen, Spasmen und Ruhigstellung kam. Elf lebensgeschichtliche Interviews mit ehemaligen Bewohner:innen ergänzen die Quellenbasis der publizierten Arzneimittelstudien und Krankenakten sowie Verwaltungsakten der jeweiligen Einrichtungen sinnfällig und würdigend. Die Bochumer Professorin für theologische Ethik Katharina Klöcker hat die von Kaminsky dargelegten Informationen einer ethisch-moralischen Beurteilung unterzogen, für die sie eigene Kriterien ausformuliert. Diesen zufolge sind die Motivation für sowie die Überzeugung von der Richtigkeit einer Behandlung für deren Folgen maßgeblich. Demnach wurde durch die Decentan-Erprobungen an Kindern in einer Fürsorgeeinrichtung diesen ein moralisch nicht zu rechtfertigendes Leid zugefügt.

Die dritte hier näher betrachtete Studie haben die von Bodelschwinghschen Stiftungen Bethel in Auftrag gegeben. Sie wurde von Niklas Lenhard-Schramm, Maike Rotzoll und dem emeritierten Heidelberger Kinderneurologen Dietz Rating erstellt. Die Arbeit beleuchtet den sozialen Kontext der jungen Bundesrepublik und die rechtlichen Rahmenbedingungen, die bis zum Inkrafttreten des Arzneimittelgesetzes vage waren, sowie die ethische und praktisch-professionelle Dimension. Die Institution Bethel wird in ihrer Eigenheit und Spezifität ausführlich beschrieben und durch viele Einzelfallbeispiele und einen Bildteil eindrücklich plastisch und lebendig dargestellt. Für die Ergründung der Arzneimittelprüfung wurde auf Verwaltungs- und Krankenakten der von Bodelschwinghschen Stiftungen und auf korrespondierende Dokumente in den Archiven der pharmazeutischen Industrie zugegriffen. Am Einzelbeispiel der Epilepsieforschung wird das zentrale Problem deutlich. Die in Bethel arbeiteten Ärzt:innen verknüpften mit der Erprobung von neuen Arzneimitteln gegen Epilepsie dreierlei: Sie wollten ihren Patient:innen Heilung beziehungsweise Linderung verschaffen, ihre eigene wissenschaftliche Expertise verbessern und der Institution Bethel durch die Zusammenarbeit mit der Industrie wirtschaftliche Vorteile ermöglichen. Dafür wurden auch minderjährige, in ihrer Entscheidungsfähigkeit beeinträchtigte Patient:innen in der Arzneimittelerprobung miteinbezogen. Ihre aufgeklärte Einwilligung wurde damals nicht als notwendig erachtet, eine schriftliche Zustimmung der Eltern oder Erziehungsberechtigten findet sich in den Akten nicht dokumentiert. Für die diagnostische Pneumenzephalographie war ein solches Dokument hingegen notwendig (Roelcke 2019). In Bethel, wo eine theologische Leitung mit der diakonischen Begleitung und leitenden Ärzt:innen zusammenkam, entwickelte sich die medizinische Forschung im Rahmen einer „Klinifizierung“ der Einrichtung. Die von Lenhard-Schramm/Rating/Rotzoll untersuchte Stichprobe von 265 Patient:innenakten umfasst etwa 10 Prozent aller infrage kommenden Akten von minderjährigen Patient:innen für den Zeitraum von 1949 bis 1975. Ausführliche und farblich abgesetzte Diagramme stellen leicht fasslich dar, wie die Stichprobenziehung durchgeführt wurde und wie sich die minderjährige Stichprobenpopulation zusammensetzte. Insgesamt fanden die Autor:innen 55 Arzneimittelprüfungen mit Antiepileptika, die entweder noch nicht auf dem Markt waren (auch damals war eine Ersterprobung von Psychopharmaka an Kindern ethisch nicht zu rechtfertigen) oder nur eine Zulassung für den Markt außerhalb Deutschlands erhalten hatten. 29-mal wurde eine Arzneimittelprüfung mit einem psychopharmakologischen Prüfpräparat durchgeführt. In diesem Zusammenhang fiel ebenfalls auf, dass Patient:innen häufig mit sedierenden Psychopharmaka behandelt wurden. Der Verdacht erscheint plausibel, dass Dämpfungsmittel gegen Unruhe verabreicht wurden, um die Stimmung im Haus beziehungsweise auf der Station zu beruhigen.

Auch in der Schweiz mit ihren global operierenden Pharmakonzernen wurde zu Arzneimittelversuchen geforscht. Allerdings legen Marietta Meier, Mario König und Magaly Tornay den Schwerpunkt ihrer Untersuchung hierzu anders als die bereits beschriebenen Studien, nämlich auf die Forschung an chronisch Erkrankten. Für die historische Erkundung der klinischen Versuche in der Psychiatrie Münsterlingen, Kanton Thurgau konnten die Bearbeiter:innen auf den Nachlass des Psychiaters und Anstaltsleiters Roland Kuhn (1912–2005) zugreifen. Er hatte ihn noch zu Lebzeiten geordnet und sortiert und mit Hinweisen für die Nachwelt markiert. Den Autor:innen dieser Studie ist durch die Hinzuziehung komplementierender Archivalien wie Krankenakten, Verwaltungsakten und auch Akten aus dem Archiv der pharmazeutischen Industrie gelungen, ein besonders facettenreiches Bild zu zeichnen. Weil sie nicht auf der rein aufdeckenden Ebene der Menschenversuche ohne Einwilligung verharren, gelingt ihnen eine ertragreiche Durchdringungstiefe des gesichteten Materials. Sie klassifizieren die Versuchspersonen nicht nach Alter, da sie ihnen einschließlich der ambulant Versorgten als vulnerabel gelten. Zudem machen sie auf eine Gruppe aufmerksam, die in deutschen Einrichtungen bislang nicht betrachtet wurde: Kuhn hatte auch Pfleger:innen der Anstalt zu Proband:innen gemacht. Ihre doppelte Abhängigkeit wird von den Autor:innen gut herausgearbeitet. „Bei manchen Angestellten führte schon die Klinikhierarchie zu Abhängigkeits- und Ohnmachtsgefühlen“ (Meier et al. 2019: 106). Kuhn nutzte diesen Umstand bewusst, genauso wie er sich damit brüstete, keine Doppelblindstudien durchgeführt zu haben. Die Prüfpräparate in Münsterlingen waren bunt gefärbt, es wurde nicht gegen Placebo getestet, es fand keine randomisierte Gruppenaufteilung statt. Damit konterkarierte er die wissenschaftlichen Ideale in der medizinischen Forschung, die zwar proklamiert und veröffentlicht waren, indes nicht rechtlich sanktioniert wurden.

Ein erstes Fazit der Studien ist schnell gezogen: Im hier dargestellten Untersuchungszeitraum gab es keine rechtlich bindenden Regelungen. In den 1950er bis in die 1970er Jahre wurde die Gabe von Tabletten und Pillen als eine niedrigschwellige, nicht notwendig zustimmungspflichtige Intervention angesehen. Das Zeitalter des Optimismus – durch die medikamentöse Behandelbarkeit von Infektionskrankheiten mittels Antibiotika, die Beherrschbarkeit von Herzkreislauferkrankungen und das Wundermittel Cortison eingeläutet – induzierte hingegen eine „Aufbruchsstimmung der Medizin“ (Quirke & Slinn 2009: 14). Durch die Rezeptortheorie hatte man ein schlüssiges Erklärungsmodell sowohl für den Wirkmechanismus von bestimmten Substanzen als auch für die Konstruktion derselben, um eine passgenauere Wirksamkeit zu erzielen. Patient:innen-, wenn nicht gar Menschenrechte gingen in diesem Optimismus jedoch verloren. Einige Befunde – und Desiderate –, die in der Zusammenschau dieser Studien ersichtlich werden, verdienen darüber hinaus noch weitere Beachtung.

## An Arzneimittelerprobungen beteiligte Personen

Menschenrechte gelten nicht nur für Fürsorgepfleglinge und Psychiatriepatient:innen, sondern auch für das Personal dieser Einrichtungen. Das ist auch deswegen für die hier erzählten Geschichten von Bedeutung, da aufgrund von Personalmangel Medikamente zur Ruhigstellung gegeben wurden. Wie anstrengend und wohl auch krankmachend die Tätigkeit in einer psychiatrischen Anstalt oder in einem Kinderheim war, wird in den meisten Studien nur am Rande angesprochen. Die Autor:innen von *Testfall Münsterlingen* schlagen hier eine bislang verdeckte Blickschneise frei. Ihre Perspektive geht tiefer als der Blick auf Leid und Unrecht, weil sie gerade die möglichen Aktionsräume wahrnehmen wollen. So schreiben sie:

„Das Konzept von Zwang und Ausbeutung ist zu eindimensional, um den Erfahrungen von Psychiatriepatient:innen gerecht zu werden […]. Dass ein Patient Prüfsubstanzen erhielt, sagt noch nicht viel darüber aus, wie er wahrgenommen wurde, wie er sich selbst sah und wie er sich vielleicht nachträglich sieht – als Opfer, als passives, reagierendes Objekt, als Subjekt, als Betroffener, Konsument oder als Akteur in einem vielschichtigen dynamischen Aushandlungsprozess, in dem psychoaktiven Substanzen und anderen Medikamenten Sinn zugeschrieben wurde.“ (Meier et al. 2019: 118)

Ärzte – hier genügt für die historischen Beispiele die männliche Form – erhielten aufgrund ihrer Stellung in den Anstalten und Einrichtungen, flankiert durch ein selten hinterfragtes paternalistisches Arzt-Patient:innenverhältnis, besondere Möglichkeiten, aktiv zu werden, sich einzubringen beziehungsweise zu profilieren. Drei Beispiele seien genannt: Uwe Kaminsky beschreibt mit Waldemar Strehl einen Anstaltsarzt, dessen Intention nicht die Heilung oder Behandlung im Sinne einer Linderung von psychiatrischen Symptomen war. Vielmehr ging es ihm vordergründig um eine „Verbreiterung der pädagogischen Angriffsfläche und Schaffung der Voraussetzung für eine gezielte Psychotherapie“ (Wagner 2020: 198).

Der Kinder- und Jugendpsychiater Hans Heinze jun. (1923–2012) ließ in den Rotenburger Anstalten der Inneren Mission eine Versuchsreihe mit Encephabol (Pyrithioxin) an Kindern mit geistiger Behinderung durchführen (ebd.: 125). Als sich Heinze Ende der 1960er Jahre auf die Stelle des Kinder- und Jugendpsychiaters im Stephansstift Hannover bewarb, betonte er in der Bewerbung seine Publikationen auf dem Gebiet der Psychopharmakologie (Winkler & Schmuhl 2019: 399). Später wechselte er zuerst nach Wunstorf und danach als Psychiatriereferent ans Sozialministerium Niedersachsen. Seine Forschungen wurden in dem Bericht von Sylvelyn Hähner-Rombach und Christina Härtel (2019) profund aufgearbeitet. Kritik an Heinzes Forschungsdesign wurde erst nach seinem Tod geäußert, zu seinen Lebzeiten war er anerkannt.

Somit stellt sich die Frage, ob die besondere Situation von Anstaltsärzt:innen noch stärker erforscht werden sollte. Hier wäre es lohnend, genauer auf die Konstellation innerhalb der Personalgefüge im Untersuchungszeitraum zu blicken. Gab es so etwas wie kollegiale Kontrolle, Teamarbeit und Absprachen oder agierten die Ärzt:innen als Anwendungsforscher:innen allein auf weiter Flur, konnten sich als Einzelkämpfer inszenieren und wurden auch so gesehen? Eine mögliche These wäre, dass sich eine selbstherrliche Praktik entwickeln konnte, gerade weil Ärzt:innen die Einrichtungen der Fürsorge bedingt durch schlecht ausgebildetes oder zu wenig Personal ohne kollegiales Abgleich- oder Kontrollinstrument allein betreuten. Doch wäre eine solche Austauschmöglichkeit nicht ein wichtiges Kontrollinstrument – eine Leitplanke, die Moral und Ethik bestärkt hätte?

## Kinder als vulnerable Gruppe

Die Situation in der Nachkriegszeit in Deutschland war von einem unübersichtlichen Flickenteppich aus Kinderheimen, Erziehungsheimen und Verwahranstalten bestimmt. Nicht zuletzt hat die Psychiatrie-Enquête mit ihrem im Jahr 1975 vorgelegten Bericht auf das Fehlen einer adäquaten Betreuung für psychisch kranke Kinder aufmerksam gemacht: Entwicklungsgestörte und Kinder mit geistiger Behinderung wurden demzufolge gemeinsam mit psychisch Erkrankten in Anstalten verwahrt; es fehlte an qualifiziertem Pflege- und pädagogischem Personal oder Sozialarbeiter:innen. Die Kinder- und Jugendpflege war geprägt von mangelhafter Versorgung, fehlenden Rückzugsräumen und einem permanenten Hin- und Herwechseln der Zuständigkeit (Borck & Lingelbach 2023). Als Anlass für eine medikamentöse Behandlung war nicht nur die Veränderung beziehungsweise Verbesserung des Wohlbefindens des einzelnen Kindes entscheidend, sondern – in der Ära nach dem Volkskörper-Denken – auch die Absicht, auf einer Station oder in einem Bettensaal Ruhe und Ordnung herbeizuführen. Der Wert eines Kindes, die Bedeutung eines Beziehungsnetzes sowie die Auffassung von der Individualität eines heranwachsenden Menschen unterschieden sich in der Nachkriegszeit deutlich von der Jetztzeit. Daher gilt es, hier genau zwischen dem Familienalltag in den 1950er und 1960er Jahren und dem Alltag in einer Einrichtung zu vergleichen und den Bezugsrahmen nicht allzu verzerrt anzulegen.

## Orte und Räume

Nicht nur in Psychiatrien und Landesheilanstalten wurden Medikamente verabreicht, sondern auch in Einrichtungen der Erziehungsfürsorge. Die Abgrenzung zwischen diesen Einrichtungen – Fürsorgeheim, Erziehungsanstalt, jugendpsychiatrische Einrichtung – war nicht sehr scharf, gleichwohl trifft der Begriff der „totalen Institutionen“ nicht zu (Wagner 2020: 26; Beyer et al. 2021: 18). Er fokussiert nämlich auf den Opferstatus, indem er die rigiden Organisationsabläufe betont und therapeutische Ansätze, die nicht auf Beibehaltung, sondern auf Veränderung abzielten, eher in den Hintergrund verschiebt. Das Personal war jedoch auch betroffen und involviert; seine Handlungsspielräume können durch die Betonung einer totalitären Institution nur unvermögend erfasst werden. Diese Räume waren (und sind) ambivalent, denn die Intention der Fürsorgeerziehung bestand zunächst in der Verwahrung – einer Herausnahme aus der Gesellschaft aus Gründen, die vom bürgerlichen Ideal bestimmt wurden. Für Toleranz gegenüber einer Andersartigkeit war in den Zeiten des Wirtschaftswunders kein Platz. Dies wird im Bericht von Christoph Beyer et al. über Schleswig-Holstein prägnant deutlich (Beyer et al. 2021: 75).

Die häufige Trägerschaft durch die Diakonie oder die Innere Mission ermöglichte starke Einfluss- und Steuerungsmöglichkeit vonseiten der Kirchen: Katholische Einrichtungen der Fürsorge für Schwachsinnige beziehungsweise Menschen mit geistiger Behinderung, der diakonisch geführte Wittekindshof, die Rotenburger Anstalten (s. Wagner) oder die von Bodelschwinghschen Anstalten in Bethel-Bielefeld wurden von den jeweiligen Landesregierungen weder ausreichend überwacht noch finanziert – ein Phänomen, das bereits seit dem Kaiserreich bestand, bekannt war sowie wiederholt beklagt wurde.

Eine Geschichte der Arzneimittelerprobungen in der Nachkriegszeit ist auch eine Geschichte der Medikalisierung des Anstaltsbetriebs: Medizinisch geschultes Personal wurde verstärkt eingestellt, Therapieansätze veränderten sich. In den Beschreibungen der vorliegenden Störungen wurden vermehrt Begriffe aus der medizinischen Fachsprache verwendet – deviantes Verhalten wurde als Psychopathie bezeichnet und so für den medizinischen Zugriff erschlossen. Zugleich herrschte in der direkten Nachkriegszeit die Forderung nach Ordnung, unbedingtem Gehorsam durch starke Reglementierung sowie eine Leibfeindlichkeit und eine erforderte Keuschheit vor. Dementsprechend wurden Kinder und Jugendliche als aufsässig, aufmüpfig und verwahrlost beschrieben, nur weil sie sich eben nicht in diesen strengen Ordnungsrahmen einpassen konnten oder wollten. Fürsorgezöglinge waren damals der Anstaltsleitung und dem Personal, welches unter schlechten Bedingungen mit schlechter Ausbildung arbeitete, schutzlos ausgeliefert.

Die Medikalisierung brachte nun einen neuen Interessensimpuls in die Kinder- und Jugendheime und auch in die Psychiatrie. Es ging um den Erhalt der Ordnung in der Anstalt, um die Erziehung und die Fügsamkeitsmachung des einzelnen Fürsorgezöglings und um eine Vergrößerung der Zugriffsfläche auf die einzelnen als pathologisch beschriebenen Kinder und Jugendlichen. Hier bot sich die Medizin mit ihren als niedrigschwellig angesehenen Interventionen im Bereich der medikamentösen Behandlung an.

## Ausblick

Die hier besprochenen Studien zeigen, dass interdisziplinäres Vorgehen unter Zugriff auf Archivalien aus Anstalts‑, Industrie- und Verwaltungsarchiven durch Zeitzeug:inneninterviews und Einsichten in private Nachlässe sinnfällig ergänzt werden kann. Weil die Aufgabe einer Patient:innenakte nicht etwa darin lag, Informationen für die historische Nachwelt, sondern primär für die am nächsten Tag arbeitenden Kolleg:innen festzuhalten, finden sich dort nur Spuren von Arzneimittelerprobungen. Deren korrespondierende Unterlagen in den Archivalien der Anstaltsverwaltung oder auch bei den Pharmafirmen müssen aktiv gesucht werden. Erst die sondierende und spezifische Analyse von Sylvia Wagner brachte das Unrecht zutage. Doch was kommt nach dem Aufdecken? Die Entschuldigung der Institutionen. Und was kommt danach? Eine Klärung, wie solche Zustände verhindert werden können oder wie solche toxischen Interessenkollisionen frühzeitig erkannt werden und ihnen dann von gegebener Stelle Einhalt geboten werden kann, denn Mitverantwortung muss in einer gelebten Demokratie insbesondere an ihren Grenzen aktiv gelebt werden. Zumal solches Handeln – und das zeigen die herangezogenen Publikationen an ganz unterschiedlichen Punkten – auch nach den damaligen ethischen Kriterien als Grenzüberschreitung zu sehen war. Die Reichsrichtlinien von 1931 und der Nürnberger Kodex von 1949 wurden ausgeblendet oder vielmehr nicht berücksichtigt – weder was die Einwilligung noch was die Proband:innenauswahl betraf. Kinder waren nicht einwilligungsfähig und galten damit zwar als besonders schützenswert, doch Heimkinder waren von dieser Vulnerabilität ausgenommen. Es wurde mit zweierlei Maß gemessen, was medizinische Interventionen betraf: Für die Durchführung einer Pneumenzephalographie oder eines EEG wurde eine schriftliche Einwilligung der Erziehungsberechtigten verlangt, Medikamentengaben waren davon allerdings nicht tangiert. Rechtliche Regelungen – die in diesem Review allerdings nicht im Fokus stehen – wurden erst mit dem Gesetz zur Neuordnung des Arzneimittelrechts 1976 verabschiedet.

Wissenschaftshistorisch spannend wäre eine Forschung über die persönlichen und Institutionengeschichten sowie die sozial- und gesellschaftshistorischen Analysen hinaus. Die historiografischen Studien zur Geschichte der Arzneimittelerprobung nach dem Nationalsozialismus detektieren wie ein Seismograf die Stimmung, die Atmosphäre, die Arbeits- und Lebensbedingungen sowie das übergeordnete Ziel der jeweils untersuchten Einrichtungen. Sie folgen dem Ansatz der Institutionengeschichte. Die Medikamentenerprobung ohne klar umrissene klinische Indikation im Sinne einer medizinischen Diagnose ist ein Gebiet, das in der Arzneimittelgeschichte bislang kaum betrachtet wurde. Als Ausnahmen zu nennen sind Balz (2010) und Tornay (2016). Eben dieser Punkt scheint für den wissenschaftshistorischen Erkenntnisgewinn am ertragreichsten. Für wie verlässlich wurden die Daten aus Arzneimittelerprobungen in Einrichtungen der Kinder- und Jugendfürsorge angesehen? Ab wann wurde nach der Reliabilität der Dokumentation, der Durchführung sowie der Sorgfalt bei der Proband:innenauswahl gefragt? Wo gab es Möglichkeiten der Abweichung vom Prüfprotokoll oder der Verweigerung aufseiten der „Proband:innen“, beim involvierten Pflegepersonal oder auch bei den durchführenden Ärzt:innen? Wurde die Medikalisierung der pädagogischen Fachsprache reflektiert? Wurde die Güte dieser an Heimkindern durchgeführten Versuche hinsichtlich ihres wissenschaftlichen Erkenntniswerts kritisch diskutiert? Wäre dies nicht die Möglichkeit, die aktuellen Anwendungsbeobachtungen in der Medizin auf ihren wissenschaftlichen und klinischen Wert zu hinterfragen und hier eine Geschichte derselben in Zeiten von evidenzbasierter Medizin anzustoßen?

Fachpolitisch erscheint es vordergründig als positive Entwicklung, dass viele Mikrostudien zu einzelnen Heimen und Anstalten durchgeführt wurden und weitere nach dem nun vorgelegten Schema auf den Weg gebracht werden können. Um hier nicht einem Klein-Klein mit nur noch lokalem Erkenntnisgewinn eines „we too“ anheimzufallen, ist es geboten, die für eine epistemologische Reflexion notwendigen interdisziplinären Zugänge zu gewährleisten.
